# Editorial: Eco-friendly synthesis & antibacterial applications of metal nanoparticles

**DOI:** 10.3389/fbioe.2023.1157374

**Published:** 2023-02-22

**Authors:** Md. Amdadul Huq, M. Mizanur Rahman, Priyanka Singh, Shahina Akter

**Affiliations:** ^1^ Department of Food and Nutrition, College of Biotechnology and Natural Resource, Chung-Ang University, Anseong, Republic of Korea; ^2^ Department of Biotechnology and Genetic Engineering, Faculty of Biological Science, Islamic University, Kushtia, Bangladesh; ^3^ The Novo Nordisk Foundation, Center for Biosustainability, Technical University of Denmark, Lyngby, Denmark; ^4^ Department of Food Science and Biotechnology, Gachon University, Seongnam, Republic of Korea

**Keywords:** eco-friendly synthesis, antibacterial applications, metal nanoparticles, Editorial, multidrug-resistant bacteria

Nanoparticles have attracted a great deal of attention across different fields of science, including, biotechnology, biomedicine, engineering, and chemistry (Huq et al., 2022a). Metallic nanoparticles include materials such as gold, silver, cobalt, copper (II), iron, lithium, magnesium, zinc, etc. The core features of these nanomaterials are their modification capability, polarity, and property diversity. Therefore, the application of these nanoparticles in the biomedical field offers many revolutionary solutions in the development of multi-functionalized drugs and products with antibacterial properties. Different physical and chemical methods have been applied for the synthesis of metal nanoparticles. However, both physical and chemical methods have many drawbacks, including the use of toxic chemicals, the synthesis of harmful byproducts, they require enormous energy and pressure, etc. (Akter and Huq, 2020; Huq et al., 2022a). Therefore, the development of a green approach for the eco-friendly, rapid, and mass synthesis of nanoparticles is an emerging demand to avoid the drawbacks of conventional physical and chemical methods.

The emergence of multidrug-resistant bacteria is a major concern for public health. Antibiotic-resistant microorganisms cause life-threatening diseases in humans. The development of novel antibacterial agents is the key solution to this issue. Therefore, eco-friendly synthesized metal nanoparticles could be promising agents to control these multidrug-resistant bacteria (Huq, 2020; Huq and Akter, 2021). Various biological resources such as plants or plant products, and microorganisms such as bacteria, fungi, and algae could be used for the ecofriendly, mass, easy, and rapid synthesis of nanoparticles (Huq et al., 2022a; Huq et al., 2022b). The synthesized metal nanoparticles could be utilized as antibacterial agents to control various pathogenic bacteria. This Research Topic is focused on the eco-friendly synthesis and antibacterial applications of metal nanoparticles.

One review article in this Research Topic described different methods of green synthesis of various metal nanoparticles such as silver, gold, zinc, etc., using plants, fungi, bacteria, and algae (Chopra et al.) The authors also described various applications of green synthesized nanoparticles in different fields of science (Chopra et al.) Three original articles on this Research Topic are devoted to the green synthesis of metal nanoparticles and nanobioconjugates and the evaluation of their antibacterial properties. Wang et al. investigated the green synthesis of silver nanoparticles (AgNPs) and discovered their potent antimicrobial activity against methicillin-resistant *Staphylococcus aureus*. Their study suggested that the synthesized AgNPs could be used as an excellent new-type drug for wound treatment infected with multidrug-resistant bacteria. Geremew et al. reported the biosynthesis of AgNPs using the plant extract of *Rumex nepalensis* and investigated their bactericidal effect against different food-borne pathogens. This study demonstrated that the biosynthesized AgNPs exhibit strong antimicrobial activity against *Salmonella typhimurium, Listeria monocytogenes, Escherichia coli,* and *S. aureus* and thus might be used as a new type of antibacterial agent for the treatment of multidrug-resistant foodborne pathogenic microorganisms. Muñoz et al. reported the facile method for the synthesis of Magnetite-Buforin-II-silver nanobioconjugates and discovered their strong antimicrobial activity against antibiotic-resistant bacterial strains.

We hope that the reader will find a useful reference in this Research Topic for the rapid, facile, and eco-friendly synthesis methods of different metal nanoparticles and their potential antibacterial applications.
